# A PRECEDE‐PROCEED model‐based educational intervention to promote healthy eating habits in middle school girls

**DOI:** 10.1002/fsn3.3167

**Published:** 2022-12-22

**Authors:** Asma Arshad, Fouzia Shaheen, Waseem Safdar, Muhammad R. Tariq, Muhammad T. Navid, Asma S. Qazi, Mohammad A. Awan, Muhammad W. Sajid, Humphrey K. Garti

**Affiliations:** ^1^ Quaid‐e‐Azam Medical College Bahawalpur Pakistan; ^2^ Faisalabad Medical University (FMU) Faisalabad Pakistan; ^3^ Department of Biological Sciences National University of Medical Sciences, The Mall Rawalpindi Pakistan; ^4^ Department of Food Sciences, Faculty of Agricultural Sciences University of the Punjab Lahore Pakistan; ^5^ Department of Biosciences, Faculty of Sciences COMSATS University Islamabad‐Sahiwal Campus Sahiwal Pakistan; ^6^ Department of Nutritional Sciences School of Allied Health Sciences, University for Development Studies Ghana Tamale Ghana

**Keywords:** child nutrition, dietary guidelines, eating behavior, growth, malnutrition, nutritional awareness, Pakistan

## Abstract

The present study was designed to develop Nutrition Education Program (NEP) based on PRECEDE‐PROCEED model (PPM) to address healthy eating behavior among middle school girls aged between 4 and 12 years. For this, middle school girls from grade 1 to 8 (*n* = 900) were consulted for their eating behaviors, followed by the analysis of their health problems. From 15 different schools of three large cities (Faisalabad, Lahore, and Rawalpindi) of Pakistan, students were divided into two groups: control group (*n* = 30) and intervention group (*n* = 30) from each school. The data were collected through interview‐based questionnaires according to the phases of PRECEDE Model and evaluated based on PROCEED model. Implementation of NEP was carried out through lectures. Lessons were prepared to enhance student's awareness about nutritious food and healthy lifestyle through educational pamphlets and influenced their attitude towards selection of food choices from My‐Plate. Results showed that NEP was quite successful for long‐term results. A significant increase in total caloric intake was observed after 8 weeks of NEP intervention (1694 ± 217 Kcal) as compared to before intervention (1329 ± 318 Kcal). Similarly, carbohydrate, protein, and fat content was also increased in daily diet. Conclusively, NEP based on PPM has great impact on healthy lifestyle of middle school girls. Significant difference was observed in score of health variables before and after NEP intervention.

## Graphical abstract

The present study was designed to develop Nutrition Education Program (NEP) based on PRECEDE‐PROCEED model (PPM) to address healthy eating behavior among middle school girls aged between 4 and 12 years. NEP based on PPM has great impact on healthy lifestyle of middle school girls. Significant difference was observed in score of health variables before and after NEP intervention.

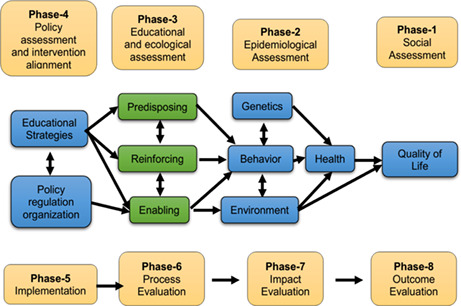

## 1 INTRODUCTION

The early childhood (aged 4–12 years) represents the largest generation of the world population, 90% of which reside in low‐ or middle‐income countries. Many studies show that dietary behaviors during early childhood contribute to the establishment of lifelong eating patterns (Alfaro et al., 2020; Aziz et al., 2018; Barasheh et al., 2017; Liu et al., 2021; Melián‐Fleitas et al., 2021; Sirasa et al., 2019; Xi et al., 2021). During the early transition period from infant to early childhood, intake of soft, semi‐solid, or solid nutrient‐dense foods is essential due to high nutritional needs. Children aged 2–5 years required adequate protein, micronutrients, and essential fatty acids marked as a significant stage for creating dietary patterns can reach out to adulthood (Motevalli et al., 2021). When children start schooling, significant weight changes are observed in them which is of public health concern. The deficiencies that are present in early age also become the health issue later in life. Healthy eating habits among these early and middle childhood stages are essential for healthy growth, cognitive development, as well as various other aspects of good physical health and mental wellbeing (Liu et al., 2021; Xi et al., 2021). A study focused on dietary habits of high school children showed that they were exposed to high‐density fast foods and their meals had vegetable and fruits in very less frequency (Alfaro et al., 2020). Their access to fast foods was much easier during school hours. The study also showed that most of them did not bring lunch to school.

In developing countries, dietary intake of school children is limited to consumption of fruits and vegetables. In Pakistan, the life style has drastically changed especially in the urban areas where the shift is seen due to lack of time among working population (Almas et al., 2020). With the sudden expansion of technology and increased production as well as ready availability of highly processed foods, have shifted preadolescent's dietary patterns from healthier to less healthy foods (Ochola et al., 2014). More intake of the processed food has been found to result in development of chronic diseases (Aziz et al., 2018). Research in the diet of children indicates that nutritional deficiency in primary school students is among the causes of low enrolment in school, high absenteeism on daily basis, early dropout, as well as poor classroom performance (Almas et al., 2020). The National Nutrition Survey (NNS) conducted by the Government of Pakistan along with UNICEF shows that 41.5% of children under the age of 5 years are underweight, 11.6% of them have wasting, of those underweight, 31% have stunted growth and about half of them are anemic (Achakzai, 2016; FAO‐UN, 2019).

Lack of knowledge about the dietary intake is of great concern in many areas of Pakistan. This lack of information leads to low nutritional intake which in turn is affecting the nutritional status especially of the individuals in the growing age. While limited resources are seen addressing this issue, intervention in the form of nutritional education by using a theoretical model increases the affectivity of the programs that make the population aware about their dietary intake (Pereira & Oliveira, 2021). PRECEDE‐PROCEED model has been used in various studies to help those health program planners that are responsible for policy making. It also helps evaluators and analyzers in different situations to design health programs efficiently. PRECEDE involves Predisposing, Reinforcing, and Enabling Constructs in Educational Diagnosis and Evaluation. PROCEED means Policy, Regulatory, and Organizational Constructs in Educational and Environmental Development (Handyside et al., 2021; Jeihooni et al., 2019; Khorsandi et al., 2012). It guides planners in comprehensive process starting with desired outcomes and going backwards to assess health and quality of life, identify strategies and design, and implement as well as evaluate health promotion programs (Marques et al., 2020; Solhi et al., 2016).

Thus, proper and targeted intervention at early stage of child's life is required. It is important to design appropriate strategies to control malnutrition and improve dietary intake to meet the requirements. The PRECEDE‐PROCEED model is a cost‐benefit evaluation framework that could be used by the health and nutrition policy makers, school administrators, and nutrition educators to analyze situations and design health and nutrition education programs effectively. The present study focused on nutrition education based on PRECEDE‐PROCEED model considering the dietary requirements of middle school students aged 4–12 years from different areas of Pakistan. The study aims to develop the nutrition education program based on healthy food choices and to assess its impact on dietary intake and knowledge of students. This study further provides a comprehensive structure for analyzing nutrition education needs of the school going children.

## 2 MATERIAL AND METHODS

### 2.1 Study design, schools, and participants

In this study, PRECEDE‐PROCEED Model (PPM) was used as a planning tool to assess the intake and nutritional knowledge before and after educational intervention. In order to educate the children, the nutrition education program consisted of lesson plans and worksheets each formulated according to the stages of PRECEDE‐PROCEED Model. For this, 15 middle schools were selected in three big cities (Faisalabad, Lahore, and Rawalpindi) of Punjab province, Pakistan, based on available facilities to implement educational lessons and no other research project was conducted in these schools related to nutritional intervention. School principals were contacted and informed comprehensively about the objectives and procedures of this study. Quasi‐experimental study was performed on 900 school girls, 60 from each school. The cluster analysis method was used for selection of children aged between 4 and 12 years if they were not participating any other research projects, were not having any clinical conditions/symptoms except malnutrition or growth impairment, and had not using vitamin and mineral supplements. Informed written consent was taken from each child's parents/guardians.

### 2.2 PRECEDE model stages

The data for the study were collected through questionnaires according to the phases of PRECEDE Model starts with diagnostic activities (social, behavioral, and educational diagnosis), predisposing factors, enabling factors, and reinforcing factors. At the stage of educational diagnosis, the predisposing, enabling, and reinforcing factors were reviewed. Predisposing factors were knowledge and diet intake of children. Enabling factors include socioeconomic status, father's occupation, family income, parent's education level, and access to the fast food. The reinforcing factors include encouragement from teachers, family, and peers. Soliciting input from key informants was cross verified from their parents/guardians and school management. The baseline data were collected for age, height, weight, and Body Mass Index (BMI). Predisposing factors were assessed by Nutrition Knowledge Questionnaire (NKQ; Table S1) consisted closed‐ended questions in which subjects were asked to either agree to or disagree with the statements about the knowledge. Enabling factors consisted of seven closed‐ended questions (Table S2). These questions included socioeconomic status, father's occupation, family income, parent's education level, and access to the fast food. For food Frequency Questionnaire (FFQ), all those behaviors were considered that were affecting the dietary intake like breakfast habits, meal skipping, and snacking according to Pakistan dietary guidelines, 2019 (Table S4). The FFQ questions were asked about the usual intake from each food group over a specified time period. Questionnaire was distributed before the start of lectures among students and was read aloud for better understanding. Students were given pretest questionnaire in the class along with the brief introduction about the purpose of researcher's study. At the end of the intervention, an immediate post‐test was conducted.

### 2.3 PROCEED model stages

The phases of PROCEED Model include impact evaluation and outcome evaluation. Evaluation was conducted after the educational intervention. A post‐test questionnaire was given to the students, which was similar to the questionnaire used in pretesting. After a time period of 1 and 3 months, a follow‐up (outcome) testing was conducted. Lectures given to the students were developed by a team of researchers and middle school teachers according to Pakistan dietary guidelines (2019) to make the intervention effective to improve the student's knowledge. Lesson plans were consisting of introduction to nutrition and balanced diet, nutrients in food (carbohydrates, protein, and fats), vitamins, minerals, My Plate, food groups (cereals, meat, dairy, fruits, and vegetables), recommended allowance from each food group, food servings, adverse effects of fast food, healthy foods choices for snacks, meal planning, physical activity, and safe food handling. The nutrition education program was a 6‐week plan with two lessons per week included PowerPoint presentations, educational posters, short films, group discussions, and question answer sessions. The time period for each lesson was 45 minutes. Worksheets were given to the students after each lecture that were prepared according to the lesson content as a reinforcing activity.

### 2.4 Data analysis

Data were managed and analyzed using Statistical Package for the Social Sciences version 25 (SPSS Inc.). Chi‐Square test was used to analyze demographic and anthropometric characteristics concerning different dietary patterns. Analysis of variance (anova) was used to evaluate statistical differences in control and intervention groups at significance *p* < .05, confidence level is 95%.

## 3 RESULTS AND DISCUSSIONS

The present study was designed to develop Nutrition Education Program (NEP) based on PRECEDE‐PROCEED model (PPM) to address healthy eating behavior among middle school girls aged between 4 and 12 years in light of Pakistan Dietary Guidelines 2019 (FAO‐UN, 2019). For this, students from 15 different schools of three large cities Faisalabad, Lahore, and Rawalpindi, Pakistan, were consulted for their eating behaviors. Numerous NEP had been set, developed, and implemented worldwide for multiple populations. However, nutritional assessment paired with instruction/lectures for promoting healthy eating habits was rarely implemented on school students. It is well understood that dynamic interplay of environmental, personal, and social behavioral factors has great impact on individual's health. More importantly, a major role played by environmental factors directly or indirectly may hinder self‐efficacy of individuals seeking healthy behaviors. We believe that an eight‐phase PPM would be helpful in creating a conceptual framework of healthy lifestyle among middle school students.

### 3.1 PRECEDE model

Prior to intervention of Nutritional Education Program through PROCEED model, four phases of PRECEDE model were used for planning of soliciting relevant information as depicted in cover figure. It provided a framework to determine nutritional/health problem in middle school girls aged 4–12 years and in formulation of educational content which addresses their needs. These systematic sequential steps of PRECEDE‐PROCEED model increase the sustainability and effectiveness of education programs intervention (Pourhaji et al., 2020).

#### 3.1.1 Social assessment (Phase‐1)

Before selection of participants, consultation was carried out with students of different grades, teaching staff, and school administrations. Demographic information for different grades was taken to understand the key issues related to health and quality of life. All of the respondents were school girls from grade one to grade eight. Data were collected through questionnaires and/or oral interviews where required. Demographic data of the participants are presented in Table 1. Cluster sampling was done for selection of participants. A total of 30 students for intervention and 30 students for control group were considered in this study from each school. Control group was taken for comparison of NEP at baseline. Participants were divided in to three subgroups based on their grade of study. Demographic data were collected for family size, birth order, parent's education, parent's occupation, and average family income.

**TABLE 1:** Demographic characteristics of middle school students

Characteristics	Intervention group *n* = 450	Control group *n* = 450	*p* value
Class of students (%)			
Group‐1 (1–3 grade)	26.7	33.3	.291
Group‐2 (1–3 grade)	33.3	33.3	
Group‐3 (1–3 grade)	40.0	33.3	
Family size (%)			
Three	24.4	22.2	.246
Four	40.0	31.1	
Five	26.7	33.3	
Six or more	8.9	13.3	
Birth order (%)			
First	35.6	26.7	.381
Second	37.8	33.3	
Third	22.2	26.7	
Fourth or higher	4.4	13.3	
Father's occupation (%)			
Govt. employee	24.4	31.1	.133
Private employee	44.4	40.0	
Business man	31.1	26.7	
Unemployed	0.0	2.2	
Mother's occupation (%)			
Employed	15.6	26.7	.116
Housewife	84.4	73.3	
Father's education (%)			
≤10th grade	8.9	15.6	.208
10th to 12th grade	13.3	11.1	
<12th grade to Bachelors	55.6	53.3	
More than Bachelors	22.2	17.8	
Mother's Education (%)			
≤10th grade	26.7	11.1	.151
10th to 12th grade	6.7	17.8	
<12th grade to Bachelors	55.6	64.4	
More than Bachelors	11.1	6.7	
Average income (%)			
<20,000	0.0	0.0	.012
31,000–40,000	4.4	0.0	
41,000–50,000	15.6	24.4	
51,000–70,000	62.2	51.1	
More than 70,000	17.8	24.4	

There was no significance difference in demographic characteristics between control and intervention group. Almost an equal number of respondents were included in both groups with respect to their grade of study. Majority of the students belong to a small family size (3–4 family members, 22.2%–40%) with moderate income status. Fathers of majority of the respondents work as private employee (40%–44.4%) followed by Bossiness man (26.7%–31.1%) in control and intervention group, respectively. Majority of the participants have educated parents. Similar pattern of participants was observed in previous researches at their own capacity (Handyside et al., 2021; Jeihooni et al., 2019; Mosavi et al., 2020; Nejhaddadgar et al., 2019; Sezgin & Esin, 2018). During application of PPM on different communities to assess their quality of life, similar strata was designed with equal capabilities of respondents (Handyside et al., 2021).

#### 3.1.2 Epidemiological assessment (Phase‐2)

In addition to demographic properties, baseline characteristics of anthropometric measures with respect to studied population were also evaluated. Results of anthropometric data are represented in Table 2. Analysis showed that there was no significant difference in baseline data of both control and intervention group. Mean age of the respondents was 9.16 ± 4.12 and 9.62 ± 5.46 years, respectively, in control and intervention group. Similarly, height weight and BMI of the respondent of both groups were statistically similar (*p* > .05). It was also observed that, on the average, all participants have poor health status (based on body mass index). Nevertheless, regarding their different grade level, health evaluation was carried out within their respective age percentile. These results suggested that quality of life of participants in both groups was analogously poor and hence, PRECEDE phase three and four needed to be applied for further planning of intervention (i.e., NEP). Similar studies were conducted previously on adult community with different heath issues needed education intervention (Franceschi et al., 2021; Labyak et al., 2021). According to dietary guideline of Pakistan, 2019, 43.9% of the young female were suffering malnutrition, especially deficiency of some minerals like iron and calcium. Anthropometric results of the present study also showed malnutrition status of middle school girls. Nutrition knowledge questionnaire was developed based on students' class and their daily life style, which is used in phase 3 of PRECEDE model.

**TABLE 2:** Mean ± SD of baseline data of middle school students

Characteristics	Intervention group *n* = 450	Control group *n* = 450	*p* value
Age (years)	9.62 ± 5.46	9.16 ± 4.12	.291
Height (cm)	135.91 ± 9.64	134.63 ± 12.4	.116
Weight (pounds)	57.91 ± 1.247	56.14 ± 1.49	.172
BMI	16.94 ± 2.99	16.98 ± 2.71	.319

Abbreviations: BMI, body mass index.

#### 3.1.3 Educational and ecological assessment (Phase‐3)

Educational Assessment was conducted according to three factors of PRECEDE model: predisposing factors, enabling factors, and reinforcing factors. For the students, the predisposing factor was knowledge and food intake. Enabling factors included socioeconomic status, father's occupation, family income, parent's education level, and access to the fast food. The reinforcing factors included encouragement from teachers, family, and peers. Educational assessment was done through nutrition knowledge questionnaire (Table S1). Questions were designed with consultation of field experts and school teachers. Generally, question responses were recorded as agree, disagree, and unsure. Questionnaire was filled by the examiners for low‐grade classes (1–5 grade); however, participants were encouraged to fill themselves. Questionnaire was prepared according to the knowledge about their daily dietary routine gained during consultation sessions with students and school staff. School cafeteria was also inspected with due permission of school administration for availability of food and food products. There was no special restriction for selling junk food such as fries, sugar drinks, samosa, burger, and shawarma, which was regularly consumed by the students during school timings. Although drinking water facility was provided by the school administration at easy access, conversely, availability of sugary soft‐drinks offers unhealthy competitive food against water.

**FIGURE 1:**
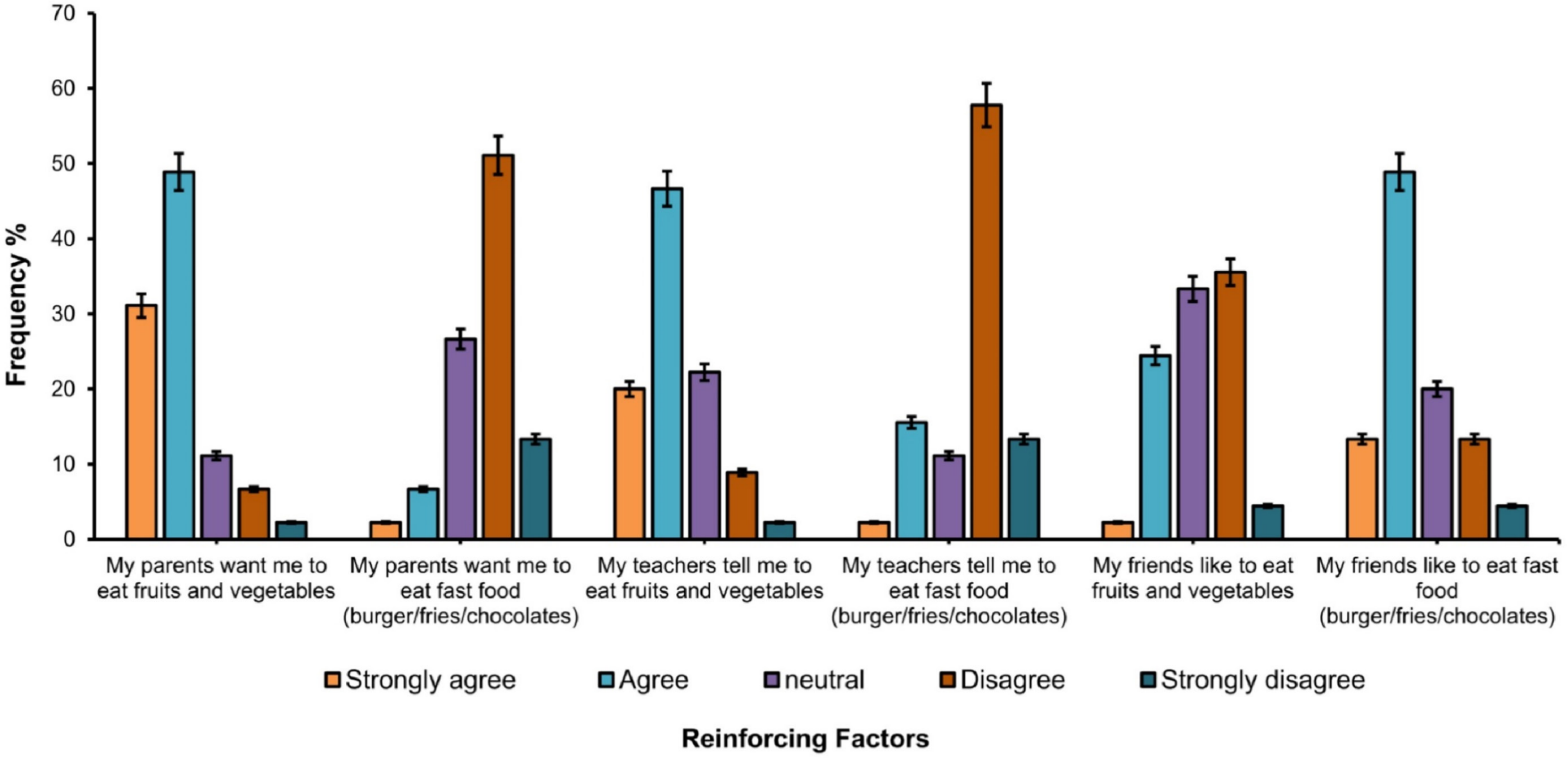
Frequency distribution (%) of reinforcing factors

Before intervention, data for knowledge and lifestyle were gathered and compared with control. No significant difference was observed between control and intervention group participants before the intervention of NEP. However, after intervention NEP, significant improvement was noticed. Student *t*‐test was performed with SPSS software to evaluate the difference in control and intervention group before NEP (Table 3). These results suggested that before intervention, students have very low knowledge about nutrition and healthy lifestyle. Similar kind of preintervention assessment was carried out on diabetic patients to maintain healthy life style (Kołota & Głąbska, 2021). The selected participants had same nutritional knowledge, attitude, and enabling factors. In another study on development of culinary nutrition knowledge among school students, analysis on intervention and control group was carried out together for comparison (Afrin et al., 2021; Maximova et al., 2021). Similar results were obtained in different studies conducted on different community problems (Azar et al., 2017; Handyside et al., 2021; Jeihooni et al., 2019). Six different reinforcing factors were evaluated on the grounds of five‐point scale of strongly agree, agree, neutral, disagree, and strongly disagree. Results of reinforcing factors are represented in Figure 1.

**TABLE 3:** Mean scores comparison of PRECEDE model predisposing, enabling and reinforcing factors on the bases of knowledge, attitude, and performance of the students before intervention and after interventions

PRECEDE‐PROCEED variables	Groups	Before intervention	Immediately after intervention	Four weeks after intervention	Eight weeks after intervention	*p* value *
Knowledge	Control	8.49	8.31	8.55	8.92	.0011
	Intervention	8.26	32.58	34.19	35.41	
	St. *t* test**	0.327	0.008	0.003	0.001	
Attitude	Control	18.45	19.37	18.67	18.71	.0019
	Intervention	18.41	45.69	46.11	47.30	
	St. *t* test**	0.158	0.027	0.019	0.006	
Enabling Factors	Control	23.47	23.28	23.39	23.36	.006
	Intervention	23.46	49.86	50.27	51.99	
	St. *t* test**	0.261	0.018	0.006	0.003	
Reinforcing Factors	Control	26.44	26.49	26.51	26.13	.0012
	Intervention	26.38	53.89	55.62	55.96	
	St. *t* test**	0.16	0.008	0.008	0.007	
Performance	Control	12.67	15.46	15.62	16.49	.0014
	Intervention	12.92	48.71	53.47	53.99	
	St. *t* test**	0.23	0.001	0.001	0.001	

* *p* value represents significance/nonsignificance of the resultant score for before and after three evaluations.

** Student *t* test is for comparison within control and intervention groups.

Results of reinforcing factors showed that most of the students were encouraged by their parents (approximately 80%) to eat fruits and vegetables and discourage to eat fast food which is not healthy at all. Similar results were obtained for teacher enforcement about fruits and vegetables. There is a mixed response of the students about friends eating behavior. For the question “My friend like to eat fruit and vegetables,” disagreement of the student was stronger as compared to agreement. Furthermore, most of the participants have friends who like to eat fast/cafeteria food. This unhealthy company/environment had great influence on student's health. (Jeihooni et al., 2019) also reported direct influence of friend circle and social pressure on eating behavior of school going students. However, after holding 3 months educational sessions for teachers and parents and also presenting educational content to social network groups enhanced score of reinforcing factors. Peer groups in schools directly effect on choosing and continuing lifestyle patterns (Pourhaji et al., 2020). Overall results of reinforcing factors were in accordance with the anthropometric results. Malnutrition status of the school girls was due to unhealthy food availability and friend circle having bad eating habits. Group discussions from 251 students of elementary school, America, revealed that girls were less physically active than boys and highlighted the effect of social support on eating behavior of students (Jeihooni et al., 2019). In another study, increase in scores of reinforcing factors was observed when nutritional education was presented to parents, school officials, and teachers (Nejhaddadgar et al., 2019).

#### 3.1.4 Administrative and policy assessment (Phase‐4)

Administrative management policies were also consulted for batter health of school students. Availability of healthy food, fruits and vegetables, nuts, physical activities of students facilitated by the administration, and hygiene conditions of food area were evaluated. Data about regular eatables were collected through food frequency questionnaire (Table S3). In this last phase of PRECEDE model, availability of facilities, support, and resources were consulted for the implementation on actual intervention during PROCEED model of the study. Coordination was established with management of the schools and designated facilitators for distribution of responsibilities, budgeting, administrative barriers, personnel availability, and all other necessary supports from school, participants, and their parents. During this phase, venue of interventions/lecture sessions and timetable were also designed with consent of teachers and informants. Learning material was developed under supervision of education experts according to the objectives of this study.

### 3.2 PROCEED model

The phases of PROCEED Model included implementation of the planning during PRECEDE model, evaluation of the process of implementation, impact of the evaluation, and final outcome evaluation. Evaluations were carried out on similar grounds of knowledge, attitude, enabling factor, reinforcing factors, nutrition knowledge questions, and food frequency questionnaire assessment.

#### 3.2.1 Implementation and process evaluation (Phase 5–6)

**FIGURE 2:**
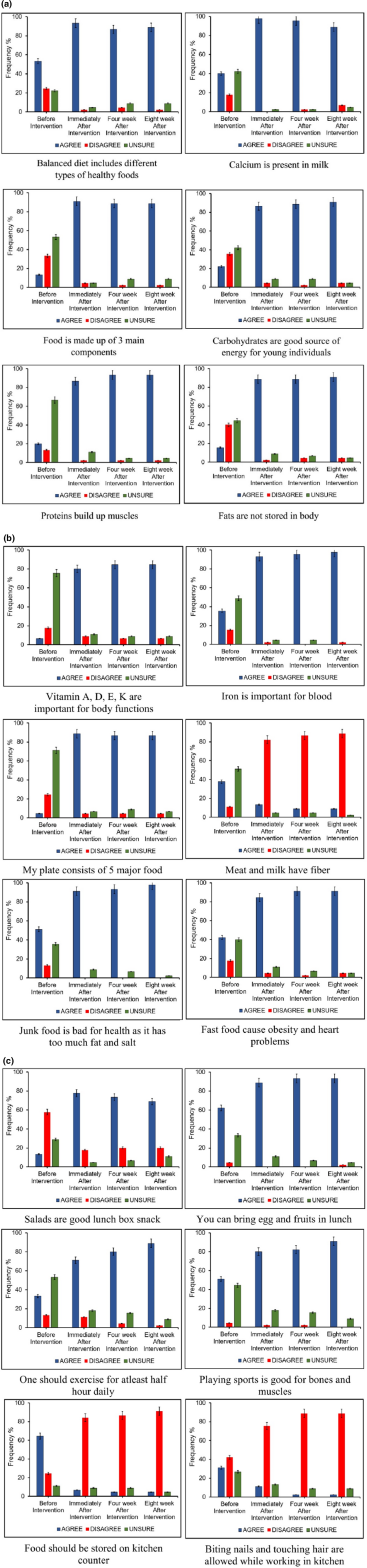
(a) Frequency distribution (%) of predisposing factors (nutrition knowledge questionnaire). (b) Frequency distribution (%) of predisposing factors (nutrition knowledge questionnaire). (c) Frequency distribution (%) of predisposing factors (nutrition knowledge questionnaire

Implementation of the nutrition education program (NEP) was carried out through lectures. Lectures were delivered by poster presentations and multimedia presentations. Content and schedule of the lectures are given in Table 4. During lectures, students exhibit enthusiasm about interesting facts for nutrition and healthy diet. After each intervention, evaluation of the process was carried out through worksheets and results are presented in Figure 2. Twelve lessons were prepared, two lessons per week, and each lesson is of 45–60 min according to the content of lesson. Knowledge assessment after interventions was compared with before intervention as well as control group. After each session, participants were encouraged to have colloquy sessions for clarity of concepts. Lessons were prepared to enhance students' awareness about nutritious food and healthy life style through educational pamphlets and influenced their attitude towards selection of food choices from My‐Plate. Students were guided about three major components of diet and their right proportion with respect to optimal calories.

**TABLE 4:** Lecture content for nutritional education program for the intervention group

Lessons	Details	Time (min)
Lesson 1	Introduction to nutrition	45
Lesson 2	Types of nutrients (carbohydrates, protein, and fats)	60
Lesson 3	Vitamins, minerals in diet	60
Lesson 4	Balanced diet	45
Lesson 5	My Plate	45
Lesson 6	Food groups (cereals, meat, dairy, fruits, and vegetables)	60
Lesson 7	Recommended daily allowance (RDA) from each food group	45
Lesson 8	Food servings	45
Lesson 9	Adverse effects of fast food	60
Lesson 10	Healthy foods choices for snacks	45
Lesson 11	Meal planning	60
Lesson 12	Physical activity and safe food handling	60

A special session was also arranged at the end of NEP for parents and teachers to reinforce their kids/students for regular eating of fruits and vegetables and drink plenty of water. They were also guided to ask students for daily physical exercise and avoid junk/fast food from canteen. Impact of the evaluation and final outcome evaluation was carried out after 4 and 8 weeks of interventions. Numerous studies have been published on PPM of health care education; most of them evaluated outcome of the exercise immediately after a month or two (Azar et al., 2017; Azar et al., 2018; Handyside et al., 2021; Jeihooni et al., 2019; Nejhaddadgar et al., 2019; Sezgin & Esin, 2018; Solhi et al., 2016).

#### 3.2.2 Impact evaluation and final outcome evaluation (phase 7–8)

After NEP, evaluation was carried out immediately after the lessons, 4 weeks after the interventions and 8 weeks of the interventions. Results of predisposing factors including knowledge and attitude showed significant improvement after intervention (*p* = .011). Surprising, huge improvement mean score of the knowledge and attitude towards healthy diet along with intervention sessions. Highest mean score for both was observed in after 8 weeks of intervention. While comparing with control group (8.92), significant improvement in knowledge was observed in intervention group (35.41). Similar results were noticed in attitude variable. Comparing with control group (18.71) significant improvement (*p* = .0019) in attitude was observed in intervention group (47.30). The mean score of the enabling factors also shows significant improvement (*p* = .006) towards healthy diet along with intervention sessions. Highest mean score was observed after 8 weeks of intervention (51.99) as compared to before intervention.

While comparing with control group (23.36), significant improvement in enabling factors was observed in intervention group (51.99). Mean score of the reinforcing factor towards healthy diet along with intervention sessions also showed significant difference (*p* = .0012). Highest mean score was observed after 8 weeks of intervention. While comparing with control group (26.13), significant improvement in knowledge was observed in intervention group (55.96). Similar results were noticed in attitude variable. Similarly, highest mean score was observed in performance after 8 weeks of intervention. While comparing with control group (16.49), significant improvement in knowledge was observed in intervention group (53.99). Similar results were noticed in attitude variable. These results showed that NEP based on PPM has high impact on middle school girls. Although in advanced countries PPM was used by many researchers, a very few interventions were reported in developing countries (Pourhaji et al., 2020). Results from previous studies showed desirable general health status and quality of life score by similar strategies of PPM. However, (Pourhaji et al., 2020) presented relatively unfavorable quality of life scores while using PPM‐based educational intervention. In several recent studies, significant increase was observed in knowledge, attitude, and of self‐efficacy scores of intervention group by PPM (Handyside et al., 2021; Jeihooni et al., 2019; Lee & Lee, 2020; Nejhaddadgar et al., 2019).

Comparative analysis of predisposing factors is presented in Figure 2b,c. Significant difference was observed before and after intervention. However, there was no significant difference immediately, after 4 weeks and after 8 weeks of intervention. These results showed that NEP was quite successful for long‐term results. Nutrient intake was also calculated from food frequency questionnaire. Results are depicted in Tables 5 and 6. Total caloric intake before intervention was 1329 ± 318 Kcal; after 8 weeks of NEP intervention (1694 ± 217 Kcal), significant difference was observed in total calories. Similarly, carbohydrate, protein, and fat content was increased in daily diet.

**TABLE 5:** Nutrients intake (mean ± SD) calculated by food frequency questionnaire (FFQ)

FFQ	Before intervention	After intervention
Energy (Kcal)	1329 ± 318	1694 ± 217
% Energy from fat	22.1 ± 3.47	29.8 ± 1.19
% Energy from proteins	10.7 ± 1.14	15.4 ± 1.62
% Energy from carbohydrates	67.2 ± 12.9	54.8 ± 6.71
Carbohydrates (g)	223.6 ± 6.38	230.6 ± 11.6
Proteins (g)	35.5 ± 1.07	65.2 ± 8.71
Fat (g)	32.8 ± 2.91	51.9 ± 3.95
Fiber (g)	8.12 ± 2.66	18.5 ± 2.67
Calcium (mg)	418.55 ± 68.4	894.1 ± 37.9

**TABLE 6:** Dietary intake based on food frequency questionnaire (FFQ)

Sr. no	Foods	Weekly (%)				Daily (%)		
		0–1	2–3	4–6	>6	0–1	2–3	4–6
1	Chappati	2	11	24	62	9	78	13
2	Rice	44	38	18	0	87	13	0
3	Pasta	64	24	11	0	93	7	0
4	Bread/toast	16	18	27	40	98	2	0
5	Pulses	33	47	20	0	87	13	0
6	Beans	64	31	4	0	91	9	0
7	Mutton	64	24	11	0	98	2	0
8	Chicken	78	22	0	0	98	2	0
9	Egg	7	62	13	18	76	24	0
10	Milk	20	24	24	31	64	36	0
11	Fruits	24	20	31	24	73	27	0
12	Vegetables	60	36	4	0	93	7	0
13	Nuts	76	24	0	0	100	0	0
14	Burger	84	16	0	0	100	0	0
15	Sandwiches	53	36	11	0	100	0	0
16	Shawarma	80	18	2	0	100	0	0
17	Samosa	40	16	31	13	87	13	0
18	Nuggets	22	42	22	13	91	9	0
19	Fries	47	18	16	20	96	4	0
20	Juices/cold drinks	18	40	22	20	89	11	0
21	Packaged snacks	29	40	13	18	80	20	0
22	Chocolate	49	31	11	9	91	9	0
23	Biscuits	36	40	11	13	96	4	0
24	Milo	80	11	4	4	100	0	0

Conclusively, NEP based on PPM has great impact on healthy lifestyle of middle school girls. Significant difference was observed in score of health variables before and after NEP intervention. Previous studies also show successful application of this model for health education programs on different communities. Results from present study showed that school going students especially girls have nutritional deficiencies. Furthermore, they have unrestricted access to junk and fast food with unbalanced dietary components, high fat content, and even unhygienic servings. During this study, school administration was suggested for abiding by these processed foods and to facilitate students with fresh fruits and vegetables. Similarly, easy access of sugar beverages limits water consumption in school students. Therefore, students should be encouraged by the parents, teachers, and school administrations adopting healthy lifestyle. PPM‐based nutrition education can be effective in reducing malnutrition in school going students. PPM improvisation, according to the participants, was often required to reduce potential barrier in successful outcomes. Findings of this study can be used to improve healthy eating behavior across populations among all age groups. Especially, PPM would be very effective as a primary intervention in school going children to enhance quality of life. To maintain long‐term healthy behavior, social media approach along with face‐to‐face intervention would be a more successful way in future. Further research is required to consolidate best and easiest channels, and face‐to‐face interventions and alleviated time constraints may develop a better way to increase program effectiveness.

##### INSTITUTIONAL REVIEW BOARD STATEMENT

The current study was reviewed and approved by Institutional Review Board (IRB), Faisalabad Medical university (FMU), Faisalabad, Pakistan, and Department of Food sciences, University of the Punjab, Lahore, Pakistan. Informed consent was obtained from all participants (parents/guardians of children) involved in the study for collection and analysis of dietary data.

## CONFLICT OF INTEREST

All authors declare no conflict of interest in this manuscript.

## Supporting information


Table S1.
Table S2.
Table S3.
Table S4.

## Data Availability

The data that support the findings of this study are available from the corresponding author upon reasonable request.
