# Supplementary material for: A PRECEDE‐PROCEED model‐based educational intervention to promote healthy eating habits in middle school girls
Source: Food Sci Nutr. 2022 Dec 22;11(3):1318–27. doi: 10.1002/fsn3.3167 (PMC10002990; doi:10.1002/fsn3.3167)
Supplement: Supplementary file 1 — Table S1. Table S2. Table S3. Table S4. [file FSN3-11-1318-s001.docx]

**Title: A Precede-Proceed Model Based Educational Intervention to Promote Healthy Eating Habits in Middle School Girls**

**Running title: Educational Intervention to Promote Healthy Eating Habits**

**Asma Arshad^1^, Fouzia Shaheen^2^, Waseem Safdar^3*^, Muhammad Rizwan Tariq^4^, Muhammad Tariq Navid^3^, Asma Saleem Qazi^3^, Mohammad Ahmed Awan^2^, Muhammad Wasim Sajid^5^, Humphrey Kwesi Garti^6*^**

^1^Quaid-e-Azam Medical college, Bahawalpur, Pakistan

^2^Faisalabad Medical university (FMU), Faisalabad, Pakistan

^3^Department of Biological Sciences, National University of Medical Sciences, Abid Majeed Road, The Mall, Rawalpindi, 44000, Pakistan

^4^Department of Food Sciences, Faculty of Agricultural Sciences, University of the Punjab, Quid-i-Azam Campus, Lahore, Pakistan

^5^Department of Biosciences, Faculty of Sciences, COMSATS University Islamabad- Sahiwal Campus, Sahiwal, Pakistan

^6^Department of Nutritional Sciences, School of Allied Health Sciences, University for Development Studies Ghana, Tamale, Ghana

***Correspondence:**

Email: [hgarti@uds.edu.gh](mailto:hgarti@uds.edu.gh); [waseem.safdar@numspak.edu.pk](mailto:waseem.safdar@numspak.edu.pk)

**Table S1**

**Questionnaire: Demographic data**

Name: ____________________(optional) Age: ________________

Height:____________ Weight: ________________

BMI: _____________

**Predisposing factors**

**Nutritional knowledge questionnaire**

Following are the questions regarding nutritional knowledge. Would you agree or disagree with following statements?

|  |  | **AGREE** | **DISAGREE** | **UNSURE** |
| --- | --- | --- | --- | --- |
| 1 | Balanced diet includes different types of healthy foods |  |  |  |
| 2 | Calcium is present in milk |  |  |  |
| 3 | Food is made up of 3 main components |  |  |  |
| 4 | Carbohydrates are good source of energy for young individuals |  |  |  |
| 5 | Proteins build up muscles |  |  |  |
| 6 | Fats are not stored in body |  |  |  |
| 7 | Vitamin A,D,E,K are important for body functions |  |  |  |
| 8 | Iron is important for blood |  |  |  |
| 9 | My plate consists of 5 major food groups |  |  |  |
| 10 | Meat and milk have fiber |  |  |  |
| 11 | Junk food is bad for health as it has too much fat and salt |  |  |  |
| 12 | Fast food cause obesity and heart problems |  |  |  |
| 13 | Salads are good lunch box snack |  |  |  |
| 14 | You can bring egg and fruits in lunch boxes |  |  |  |
| 15 | One should exercise for atleast half hour daily |  |  |  |
| 16 | Playing sports is good for bones and muscles |  |  |  |
| 17 | Food should be stored on kitchen counter |  |  |  |
| 18 | Biting nails and touching hair are allowed while working in kitchen |  |  |  |

**Table S2**

**Enabling factors**

1. Who is taking care of you?

| Parents |  |
| --- | --- |
| Only mother |  |
| Only father |  |
| extended family (grandparents, uncle aunt ) |  |

1. What is the occupation of your father?

| government employee |  |
| --- | --- |
| private employee |  |
| business man |  |
| unemployeed |  |

1. Average income (rupees)

| Less than 20,000 |  |
| --- | --- |
| 21,000 to 30,000 |  |
| 31,000 to 40,000 |  |
| 41,000 to 50,000 |  |
| 51,000 to 70,000 |  |
| More than 70,000 |  |

1. Number of family members

| 3-4 |  |
| --- | --- |
| 5-6 |  |
| 7-8 |  |
| More than 8 |  |

1. father’s education level

| ≤10th grade |  |
| --- | --- |
| 10th to 12th grade |  |
| <12th grade to Bachelors |  |
| More than Bachelors |  |

1. Mother’s education level

| ≤10th grade |  |
| --- | --- |
| 10th to 12th grade |  |
| <12th grade to Bachelors |  |
| More than Bachelors |  |

1. Which of the foods are available in your school canteen?

| Burger/shawarma/sandwich |  |
| --- | --- |
| Fries |  |
| Packaged snacks |  |
| Juices and soft drinks |  |
| Chocolates and toffees |  |
| Samosa |  |

**Table S3**

**Reinforcing factors**

Please choose one option from “strongly agreed” to “strongly disagreed” according to your opinion.

|  | **Strongly agree** | **Agree** | **neutral** | **Disagree** | **Strongly disagree** |
| --- | --- | --- | --- | --- | --- |
| My **parents** want me to eat fruits and vegetables |  |  |  |  |  |
| My **parents** want me to eat fast food (burger/fries/chocolates) |  |  |  |  |  |
| My **teachers** tell me to eat fruits and vegetables |  |  |  |  |  |
| My **teachers** tell me to eat fast food (burger/fries/chocolates) |  |  |  |  |  |
| My **friends** like to eat fruits and vegetables |  |  |  |  |  |
| My **friends** like to eat fast food (burger/fries/chocolates) |  |  |  |  |  |

**Table S4**

**Food frequency questionnaire**

Please tick the option that shows that how much you consume the given foods.

|  |  | **Weekly** | | | | | | | **Daily** | | | | | | |
| --- | --- | --- | --- | --- | --- | --- | --- | --- | --- | --- | --- | --- | --- | --- | --- |
| **s** | **Foods** | **0** | **1** | **2** | **3** | **4** | **5** | **6** | **0** | **1** | **2** | **3** | **4** | **5** | **6** |
| 1 | Chappati |  |  |  |  |  |  |  |  |  |  |  |  |  |  |
| 2 | rice |  |  |  |  |  |  |  |  |  |  |  |  |  |  |
| 3 | pasta |  |  |  |  |  |  |  |  |  |  |  |  |  |  |
| 4 | Bread/ toast |  |  |  |  |  |  |  |  |  |  |  |  |  |  |
| 5 | Pulses |  |  |  |  |  |  |  |  |  |  |  |  |  |  |
| 6 | Beans |  |  |  |  |  |  |  |  |  |  |  |  |  |  |
| 7 | Mutton |  |  |  |  |  |  |  |  |  |  |  |  |  |  |
| 8 | Chicken |  |  |  |  |  |  |  |  |  |  |  |  |  |  |
|  |  | **Weekly** | | | | | | | **Daily** | | | | | | |
| **s** | **Foods** | **0** | **1** | **2** | **3** | **4** | **5** | **6** | **0** | **1** | **2** | **3** | **4** | **5** | **6** |
| 9 | Egg |  |  |  |  |  |  |  |  |  |  |  |  |  |  |
| 10 | Milk |  |  |  |  |  |  |  |  |  |  |  |  |  |  |
| 11 | Fruits |  |  |  |  |  |  |  |  |  |  |  |  |  |  |
| 12 | Vegetables |  |  |  |  |  |  |  |  |  |  |  |  |  |  |
| 13 | Nuts |  |  |  |  |  |  |  |  |  |  |  |  |  |  |
| 14 | Burger |  |  |  |  |  |  |  |  |  |  |  |  |  |  |
| 15 | sandwiches |  |  |  |  |  |  |  |  |  |  |  |  |  |  |
| 16 | Shawarma |  |  |  |  |  |  |  |  |  |  |  |  |  |  |
| 17 | Samosa |  |  |  |  |  |  |  |  |  |  |  |  |  |  |
| 18 | Nuggets |  |  |  |  |  |  |  |  |  |  |  |  |  |  |
| 19 | Fries |  |  |  |  |  |  |  |  |  |  |  |  |  |  |
| 20 | Juices/ Cold drinks |  |  |  |  |  |  |  |  |  |  |  |  |  |  |
| 21 | Packaged Snacks |  |  |  |  |  |  |  |  |  |  |  |  |  |  |
| 22 | Chocolate |  |  |  |  |  |  |  |  |  |  |  |  |  |  |
| 23 | Biscuits |  |  |  |  |  |  |  |  |  |  |  |  |  |  |
| 24 | Milo |  |  |  |  |  |  |  |  |  |  |  |  |  |  |
